# Use of an Esophageal Heat Exchanger to Maintain Core Temperature during Burn Excisions and to Attenuate Pyrexia on the Burns Intensive Care Unit

**DOI:** 10.1155/2016/7306341

**Published:** 2016-02-25

**Authors:** David Williams, Gordon Leslie, Dimitrios Kyriazis, Benjamin O'Donovan, Joanne Bowes, John Dingley

**Affiliations:** Welsh Centre for Burns, Morriston Hospital, Swansea SA6 6NL, UK

## Abstract

*Introduction*. Burns patients are vulnerable to hyperthermia due to sepsis and SIRS and to hypothermia due to heat loss during excision surgery. Both states are associated with increased morbidity and mortality. We describe the first use of a novel esophageal heat exchange device in combination with a heater/cooler unit to manage perioperative hypothermia and postoperative pyrexia.* Material and Methods*. The device was used in three patients with full thickness burns of 51%, 49%, and 45% body surface area to reduce perioperative hypothermia during surgeries of >6 h duration and subsequently to control hyperthermia in one of the patients who developed pyrexia of 40°C on the 22nd postoperative day due to* E. coli*/*Candida* septicaemia which was unresponsive to conventional cooling strategies.* Results*. Perioperative core temperature was maintained at 37°C for all three patients, and it was possible to reduce ambient temperature to 26°C to increase comfort levels for the operating team. The core temperature of the pyrexial patient was reduced to 38.5°C within 2.5 h of instituting the device and maintained around this value thereafter.* Conclusion*. The device was easy to use with no adverse incidents and helped maintain normothermia in all cases.

## 1. Introduction

Burns patients are vulnerable to hyperthermia due to sepsis and the acute inflammatory response (SIRS) to burns and to hypothermia due to heat loss during surgery for excision and grafting. Both states are associated with a significant increase in morbidity and mortality. Warming strategies can be considered in three main categories: passive rewarming where the environment is optimised (warm environment permitting endogenous heat production), active external warming (adding heat to the body surface), and active core warming (adding heat to internal body surfaces). One core warming technique in common use, including in our own institution, is the active warming of intravenous fluids in addition to use of a warming underblanket on the operating table (an external warming method). Some other external warming methods can however be problematic in a patient having a large surgical burn excision. Peripheral vasoconstriction renders active external warming less effective. Furthermore it is our experience and that of some others that active forced air warmers may actually enhance heat loss via latent heat of vaporisation of water when there are large areas of exposed wet raw tissue during surgical excision of a large burn area [1]. Despite active fluid warming, a warm underblanket, and an operating room maintained at 30°C, a passive warming technique which is very uncomfortable for staff, we and others often observe a gradual fall in patient core temperature during the course of large-area burn excisions [1–3]. Sometimes these procedures have to be performed in stages for this reason.

We describe a series of four cases in which a new esophageal heat exchange device was used to better maintain core temperature during large burn excisions and to control pyrexia during burn intensive care management.

An esophageal heat exchange device (EHED) (ECD; Advanced Cooling Therapy, Chicago, US) has recently become available for use in combination with a heater/cooler unit (Cincinnati Sub-Zero Blanketrol® II/III Hyper-Hypothermia System, Cincinnati Sub-Zero, Sharonville, Ohio, US, or Gaymar Meditherm III, Stryker Medical, Portage, Michigan, US) which circulates cool or warm water through the EHED. The esophageal heat exchange device is placed similarly to a standard orogastric tube, with additional connectors designed for standard water blanket chillers/heaters. Water circulates in a closed circuit, eliminating the risks of free water instillation into the gastrointestinal tract, while providing heat exchange via the blood circulation surrounding the esophagus. We describe here the first use of this device in a series of burned patients, as a warming device during large surface area burn excisions, followed in one instance for active cooling during postoperative fever.

## 2. Case Reports

**Procedure 1 .**

**Figure 1:**
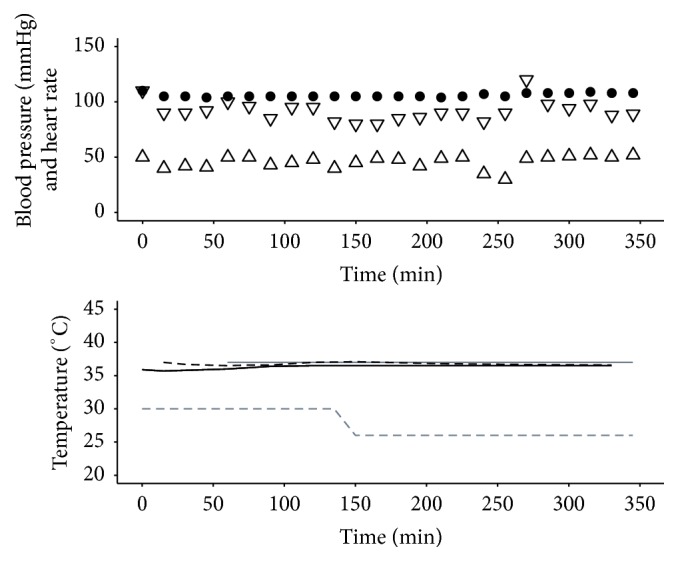
Haemodynamics and temperatures for Procedure 1. Systolic pressure (mmHg): ▽; diastolic pressure (mmHg): △; heart rate (beats per minute): ●; target temperature (°C): grey solid line; ambient temperature (°C): grey dashed line; peripheral temperature (°C): black solid line; Bladder Temperature (°C): black dashed line. The ambient operating room temperature was maintained at 30°C for the first 90 minutes of surgery, a typically high temperature for a burns operating room intended to reduce evaporative heat loss, and then reduced to a more comfortable 26°C, with no drop in patient core or peripheral temperature over the remaining four and a half hours.

A 37-year-old 60 kg male sustained smoke inhalation and 51% full thickness burns to face, chest, both legs, and arms in a house fire. He was admitted to the burns centre nine hours after initial resuscitation in another hospital and was taken to the operating room for initial burn debridement and bronchial lavage. On hospital admission day 2 the patient was taken to the operating room for staged excision of the burn wounds. An EHED was inserted alongside an existing nasogastric feeding tube by palpation to a length of 25 cm at the incisors before encountering resistance, as the face and mouth were too oedematous for insertion under direct vision by laryngoscopy. It was decided that although insertion to a greater depth might be desirable for optimal heat exchange, further efforts to achieve this would be unwise. The core temperature sensor used in this and subsequent cases was incorporated into the bladder urinary catheter (“Level 1” 14 Fr Foley Catheter with Temperature Sensor Smiths Medical ASD Inc., Rockland, MA, USA). 42% body surface area was excised and grafted during an operative time of six hours (Figure 1). During the course of this procedure the operating room temperature was reduced from 30°C, a typically elevated temperature for a burns operating room with the intention of reducing evaporative heat loss, to more comfortable 26°C, with no drop in patient core or peripheral temperature.

**Procedure 2 .**

**Figure 2:**
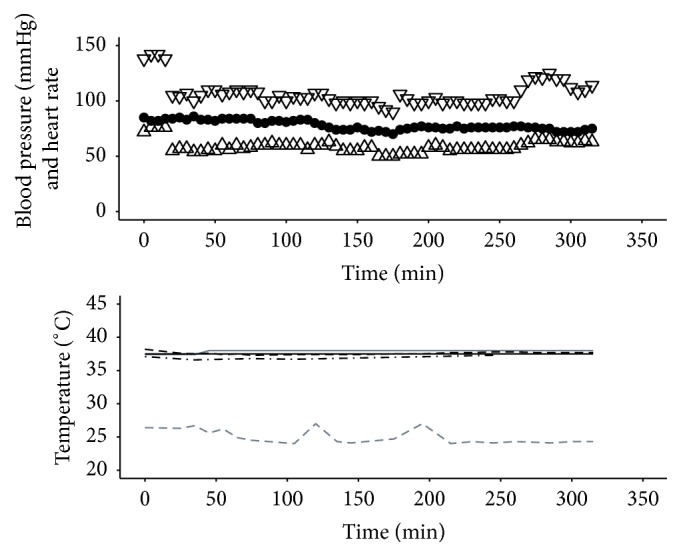
Haemodynamics and temperatures for Procedure 2. Systolic pressure (mmHg): ▽; diastolic pressure (mmHg): △; heart rate (beats per minute): ●; target temperature (°C): grey solid line; ambient temperature (°C): grey dashed line; peripheral temperature (°C): black solid line; Bladder Temperature (°C): black dashed line; Tympanic Temperature (°C): black dash-dot line. During the course of the procedure the ambient operating room temperature was reduced from approximately 26°C to 24°C while patient core and peripheral temperatures were maintained.

A 49-year-old 86 kg male sustained 49% circumferential full thickness burns to both legs and left arm and the left torso following attempted self-immolation. He was admitted to our unit 2 h after injury and taken to the operating room for escharotomies and initial debridement. On hospital admission day 2 the patient was taken back to the operating room for staged excision and grafting of the burn wounds to his legs. The nasogastric tube was temporarily removed and an EHED inserted to a length of 40 cm at the lips. 32% body surface area was excised during an operative time of six hours (Figure 2). During the course of the procedure the ambient temperature was reduced from approximately 26°C to 24°C while patient core and peripheral temperatures were maintained.

**Procedure 3 .**

**Figure 3:**
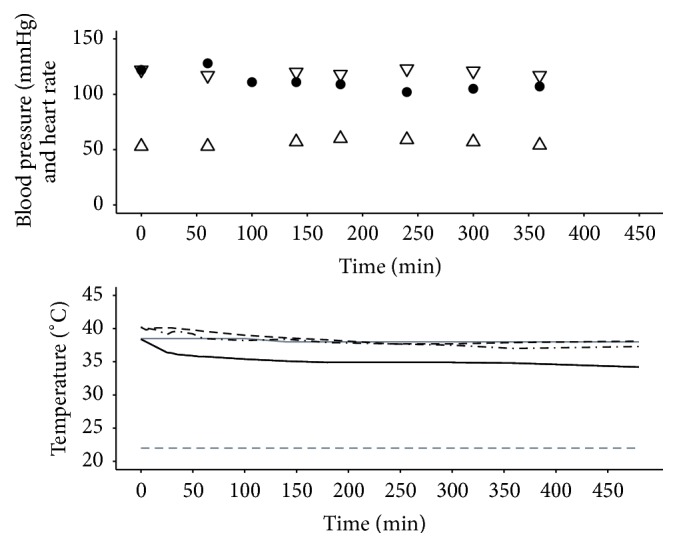
Haemodynamics and temperatures for Procedure 3. Systolic pressure (mmHg): ▽; diastolic pressure (mmHg): △; heart rate (beats per minute): ●; target temperature (°C): grey solid line; ambient temperature (°C): grey dashed line; peripheral temperature (°C): black solid line; Bladder Temperature (°C): black dashed line; Tympanic Temperature (°C): black dash-dot line. Although the temperature management unit is capable of generating a water temperature as low as 4°C, on this occasion the operator set the parameters such that the circulating water temperature did not fall below 10°C during this cooling maneuver.

By day 17, the patient in Procedure 2 had been to the operating room a further four times, and all burn wounds had been excised and grafted. On day 22 he developed pyrexia due to SIRS and* E. coli* and* Candida* septicaemia. Despite antibiotics and conventional cooling strategies (lowering the ambient temperature of the room, removing bed linen and use of cooling fans), the patient maintained a temperature of ≥40°C for six hours. Under these circumstances, we would consider continuous venovenous hemodiafiltration to assist core temperature reduction and removal of inflammatory mediators; however this intervention is highly invasive and associated with significant risks. We therefore inserted the EHED alongside the existing nasogastric feeding tube without complication and used it to effectively control the pyrexia. A target core temperature of 38.5°C was set on the attached heater/cooler unit with a minimum permitted water temperature of 12°C (Figure 3). This was a moderate setting as the heater/cooler is capable of producing a water temperature as low as 4°C.

**Procedure 4 .**

**Figure 4:**
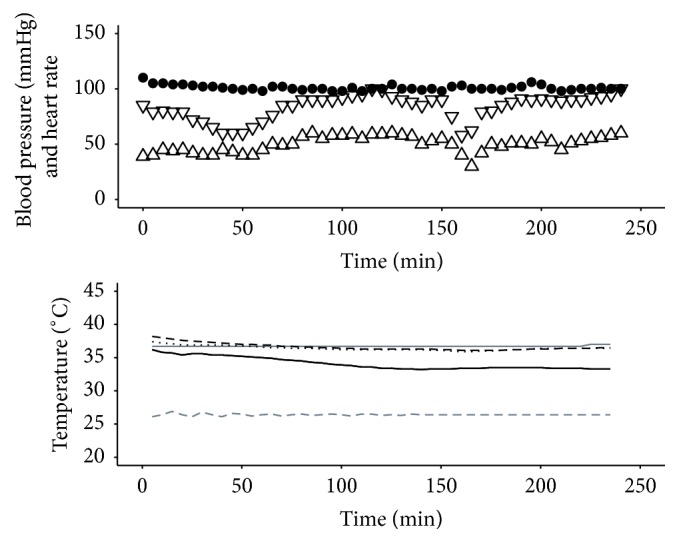
Haemodynamics and temperatures for Procedure 4. Systolic pressure (mmHg): ▽; diastolic pressure (mmHg): △; heart rate (beats per minute): ●; target temperature (°C): grey solid line; ambient temperature (°C): grey dashed line; peripheral temperature (°C): black solid line; Bladder Temperature (°C): black dashed line; nasal temperature (°C): black dotted line. Operating room temperature was maintained at a moderate 26°C for the duration of the procedure with maintenance of patient core temperature and a small drop in peripheral temperature over this period.

A 49-year-old 86 kg male (different from the patient described in Procedures 2 and 3) sustained 45% full thickness burns to left arm, abdomen, back, and groin and circumferential burns of both legs due to attempted self-immolation. He was admitted to the burns centre 10 hours after initial resuscitation in another hospital and was taken to the operating room for initial debridement, escharotomies, and tracheotomy. By hospital admission day 2 the patient was pyrexial and haemodynamically unstable, requiring inotropic support, due to SIRS. He was taken to the operating room for staged excision and split skin grafting of burn wounds. Despite conventional heat-loss prevention strategies, massive perioperative blood loss and transfusion resulted in a tendency towards hypothermia. As forced air warming was impossible due to the extent of the burns, an EHED inserted preoperatively under direct laryngoscopy to 40 cm was used to maintain normothermia. A total of 38% body surface area was excised and grafted during an operative time of six hours (Figure 4). Ambient temperature was maintained at a moderate 26°C for the duration of the procedure with maintenance of patient core temperature and a small drop in peripheral temperature over this period.

## 3. Discussion

Maintenance of normothermia (37°C ± 0.5°C) is dependent on the balance between metabolic heat production, external heating, and heat loss. The compensatory response to hypothermia is peripheral vasoconstriction and shivering while the response to hyperthermia is vasodilation and sweating.

Hypothermia causes increased mortality, morbidity, and delayed discharge from hospital. Rate of recovery from hypothermia after burn surgery is predictive of mortality [4]. Shivering and vasoconstriction increase oxygen consumption and decrease oxygen delivery resulting in tissue hypoxia which may cause myocardial ischaemia and arrhythmias, impaired wound healing, and wound infection. Hypothermia impairs platelet function, fibrinolysis, and the coagulation cascade, resulting in coagulopathy and increased transfusion requirements [5]. Pharmacokinetic effects of hypothermia include impaired drug metabolism and increased lipid solubility of volatile anaesthetic agents, resulting in delayed emergence and recovery from anaesthesia. General anaesthetic agents also impair the thermoregulatory response.

**Figure 5:**
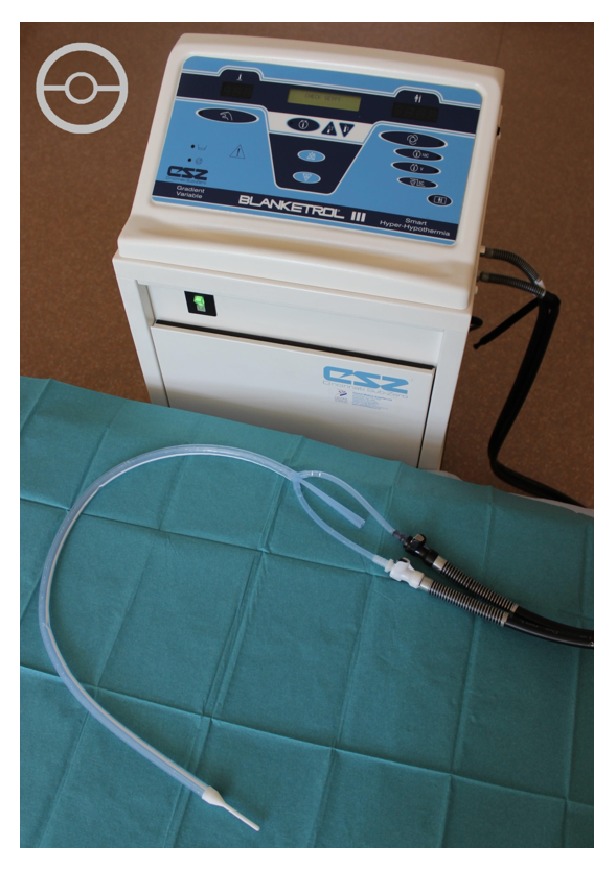
Photograph of the esophageal heat exchange device attached to the Blanketrol III temperature management unit. A cross section of the device is represented in the upper left corner.

The EHED is made of flexible silicone, is approximately 60 cm long, and is sited in the esophagus to a depth of approximately 45 cm, with the remainder left external to the mouth (Figure 5). Two lumens for water inlet and outlet connect to a heater/cooler device. A third central lumen allows gastric decompression and drainage. It is a single-use device with an intended maximum duration of use of 36 hours and is currently licensed for use in the United Kingdom, Canada, Australia, and the US.

A heater/cooler device is attached which circulates water through the EHED and uses an internal PID (Proportional-Integral-Differential) computer control program to rapidly bring patient core temperature to a user-defined set-point without overshoot. Core temperature is monitored from a thermistor sited in the nasopharynx, tympanic membrane, distal esophagus, rectum, or bladder. Our preference was to use a bladder catheter with integral thermistor as this was less likely to become displaced than a rectal probe.

Table 1 summarises the causes and conventional strategies for prevention of hypothermia.

During excision and grafting of a major burn, the extent of the injuries may preclude the use of a forced air rewarming device (e.g., Bair Hugger*™*). Warming devices which use circulating water have been shown to be more effective than forced hot air devices in maintaining temperature during burn excisions due to the greater heat capacity of water compared to air [6]. Core warming devices therefore may also have a useful role to play [7]. Intravenous infusion of hot fluids has also been described [8]. Controlling a patient's temperature through the gastrointestinal tract has been accomplished in the past with varying results. Reports have described uses both for cooling [9–12] and for warming [13–17]. It might be thought that an esophageal device would be most efficient when used to actively cool a patient as the temperature gradient between the circulating water (4°C minimum) and the patient (37°C or above) would be much greater than in a patient warming situation with a maximum circulating water temperature of 42°C and a patient temperature of perhaps 35°C. This is indeed true; however robustness of the blood flow surrounding the esophagus keeps its wall temperature much closer to body temperature, facilitating ongoing inward heat transfer even with a modest temperature gradient when used in the patient warming scenario. The internal contact surface area along a 45 cm esophagus may also seem modest; however, with a high esophageal blood flow maintaining the temperature gradient and therefore heat transfer, mathematical modelling of the process suggests a heat transfer rate into a patient of approximately 50 Watts, even when used in a patient warming role (see Appendix).

Intravenous heat exchange devices (e.g., Coolguard) haemofiltration or cardiopulmonary bypass may also be used to treat hypo- and hyperthermia; however these are highly invasive, with risks of infection, haemorrhage, and pneumothorax; and they also require systemic anticoagulation, which is associated with significant morbidity in the burned patient.

Central warming devices may reduce the need to maintain ambient temperature >29.4°C during burn excisions which can be very uncomfortable for the operating team.

Use of the esophageal route is less invasive and placement is technically quicker and easier than for intravascular devices (i.e., Coolgard or hemofilter) and suitable sites for intravascular device placement may be limited in the severely burned patient; however in one of our cases we used the EHED despite a very burned and oedematous face. The bladder is generally accessible in burns patients, and urinary catheters with integral temperature probes are readily available. Major surgical procedures at intervals of several days may require repeat placement of such warming devices and in this context we suspect the esophageal route would have a lower morbidity than repeat insertion of intravascular devices.

In the cases which underwent extensive surgical excision of burns (Procedures 1, 2, and 4), it is our clinical impression that use of the EHED helped to maintain normothermia. In the cases which were initially pyrexial (Procedures 3 and 4), use of the EHED helped to bring the core temperature down.

Major factors limiting the size and duration of excision procedures are blood loss and a gradual reduction in core temperature of the patient. If central rewarming devices improve thermal control during these procedures, one consequence is that operating time may potentially be extended; however it may still be prudent to excise and graft large burns in a staged manner, as excisions in excess of 20% in a single procedure have been associated with increased mortality [18].

Further detailed evaluations are necessary; however in all four of our cases, the EHED was easy to use, appeared to rapidly correct and maintain core temperature within normal range in both hypothermic and pyrexial patients compared to previous experience in our institution, and was less invasive than intravascular heat exchange devices. It may be useful in larger burns where the injuries preclude forced air warming and restrict central intravenous access. It may allow more extensive and prolonged surgical procedures to be undertaken without being limited by hypothermia. When using this device we were able to reduce ambient temperature to a level that was more comfortable for the operating team.

## Figures and Tables

**Table 1:** Mechanisms and prevention of heat loss.

Physical mechanism	Examples	Preventative strategies
Conduction	Operating table, cold iv, and irrigation fluids	Prewarmed iv and irrigation fluids iv fluid warmers Insulated/electrically heated mattresses

Convection	Air movement	Passive: drapes; active: forced hot air devices, circulating water devices

Radiation	Exposed tissues	Reflective blankets, drapes, and radiant heating

Phase change (latent heat of evaporation)	Evaporation from: exposed burns and surgical wounds, skin prep, and respiratory tract	Humidifiers, heat and moisture exchangers, and circle breathing systems

## Appendix

The equation that describes the heat flow of the ECD, in which heat is exchanged in a radial direction through the cylindrical configuration, is

(A.1) $$\begin{array}{c} q_{r}=2\pi kL\frac{\left(T_{i}-T_{o}\right)}{\ln\left(r_{o}/r_{i}\right)}, \end{array}$$

where *q*_*r*_ is the heat flow in Watts (W), *k* is the heat transfer coefficient for conduction through silicone (~0.4 W/m C), *T*_*i*_ is the temperature at the inner surface of the device (the temperature of the heat exchange medium) in °C, *T*_*o*_ is the temperature at the outer surface of the device (the temperature of the esophagus) in °C, *L* is the length of the device, *r*_*o*_ is the radius of the outer surface, and *r*_*i*_ is the radius of the inner surface of the device, such that *r*_*o*_ − *r*_*i*_ is the thickness of silicone through which heat transfer occurs.

The robustness of the blood flow surrounding the esophagus keeps the gradient very high and the esophageal wall temperature much closer to body temperature than perhaps might be expected, thereby further enhancing heat transfer.

Inserting reasonable values into the above equation gives the following:

*r*_*o*_ = 0.006 m,
*r*_*i*_ = 0.00535 m,
*k* = 0.4 W/m C (actually higher, but this is a conservative estimate),
*T*_*i*_ = 42°C,
*T*_*o*_ = 37°C (or colder, depending on how low patient temperature is at start),
*L* = 0.45 m (this may be more or less, depending on depth placed and therefore total contact area).

This resolves to “*q*” per length of esophageal tube of 110 W/m. Therefore with the expected contact length of 0.45 m, almost 50 W of heat would be entering the patient in the warming direction.
